# Citral alleviates an accelerated and severe lupus nephritis model by inhibiting the activation signal of NLRP3 inflammasome and enhancing Nrf2 activation

**DOI:** 10.1186/s13075-015-0844-6

**Published:** 2015-11-19

**Authors:** Shuk-Man Ka, Jung-Chen Lin, Tsai-Jung Lin, Feng-Cheng Liu, Louis Kuoping Chao, Chen-Lung Ho, Li-Tzu Yeh, Huey-Kang Sytwu, Kuo-Feng Hua, Ann Chen

**Affiliations:** Graduate Institute of Aerospace and Undersea Medicine, National Defense Medical Center, Taipei, Taiwan; Department of Pathology, Tri-Service General Hospital, National Defense Medical Center, No. 325, Sec. 2, Cheng-Gung Road, Taipei, Taiwan ROC; Graduate Institute of Life Sciences, National Defense Medical Center, Taipei, Taiwan; Division of Rheumatology/Immunology and Allergy, Department of Medicine, Tri-Service General Hospital, National Defense Medical Center, Taipei, Taiwan; Department of Cosmeceutics, China Medical University, Taichung, Taiwan; Division of Wood Cellulose, Taiwan Forestry Research Institute, Taipei, Taiwan; Department and Graduate Institute of Microbiology and Immunology, National Defense Medical Center, Taipei, Taiwan; Department of Biotechnology and Animal Science, National Ilan University, Sec. 1, Shen-Lung Road, Ilan, 260 Taiwan ROC

## Abstract

**Introduction:**

Lupus nephritis (LN) is a major complication of systemic lupus erythematosus. NLRP3 inflammasome activation, reactive oxygen species (ROS) and mononuclear leukocyte infiltration in the kidney have been shown to provoke the acceleration and deterioration of LN, such as accelerated and severe LN (ASLN). Development of a novel therapeutic remedy based on these molecular events to prevent the progression of the disease is clinically warranted.

**Methods:**

Citral (3,7-dimethyl-2,6-octadienal), a major active compound in a Chinese herbal medicine *Litsea cubeba*, was used to test its renoprotective effects in a lipopolysaccharide (LPS)-induced mouse ASLN model by examining NLRP3 inflammasome activation, ROS and COX-2 production as well as Nrf2 activation. The analysis of mechanisms of action of Citral also involved its effects on IL-1β secretion and signaling pathways of NLRP3 inflammasome in LPS-primed peritoneal macrophages or J774A macrophages.

**Results:**

Attenuated proteinuria, renal function impairment, and renal histopathology, the latter including intrinsic cell proliferation, cellular crescents, neutrophil influx, fibrinoid necrosis in the glomerulus, and peri-glomerular infiltration of mononuclear leukocytes as well as glomerulonephritis activity score were observed in Citral-treated ASLN mice. In addition, Citral inhibited NLRP3 inflammasome activation and levels of ROS, NAD(P)H oxidase subunit p47^phox^, or COX-2, and it enhanced the activation of nuclear factor E2-related factor 2 (Nrf2). In LPS-primed macrophages, Citral reduced ATP-induced IL-1β secretion and caspase-1 activation, but did not affect LPS-induced NLRP3 protein expression.

**Conclusion:**

Our data show that Citral alleviates the mouse ASLN model by inhibition of the activation signal, but not the priming signal, of NLRP3 inflammasome and enhanced activation of Nrf2 antioxidant signaling.

## Introduction

Lupus nephritis (LN), a disease characterized by immune complex-mediated renal inflammation and fibrosis, is classified into six classes according to the severity of renal histopathology [1], as transformation from a lower grade to a higher grade occurs [2, 3]. Acute induction of cellular autoimmunity [4–6] and/or humoral autoimmunity [6, 7] has been implicated in the development of the resultant severe renal conditions, although the exact pathogenic mechanisms remain to be determined. Importantly, expression of proinflammatory cytokines, including interleukin (IL)-1β [8–10] and IL-18 [9, 11], is upregulated in animal models of accelerated and severe LN (ASLN).

The NLR family, pyrin domain containing 3 (NLRP3) inflammasome is known to control the activation of caspase-1, which cleaves pro-IL-1β and pro-IL-18 to form mature IL-1β and IL-18 [12, 13]. A priming signal from pathogen-associated molecular patterns and an activation signal, e.g., ATP from damaged cells, are both required for full activation of the NLRP3 inflammasome [12–14]. Immune complexes can trigger the activation of the NLRP3 inflammasome in macrophages from systemic lupus erythematosus (SLE) patients and animal models, leading to cell and tissue damage [9, 15, 16]. We [9] and others [10, 16] have shown that increased production of IL-1β resulting from NLRP3 inflammasome activation and T cell activation occurs in mouse models of LN, suggesting the NLRP3 inflammasome and its downstream pathway as an important mechanism underlying the evolution of LN.

In addition, oxidative stress can potentiate inflammatory processes by activating NF-κB, thereby stimulating production of pro-inflammatory cytokines [17–19] and has been implicated in the progression/deterioration of LN in patients, leading to the development of severe renal conditions [17, 18, 20]. The nuclear factor E2-related factor 2 (Nrf2) antioxidant signaling pathway confers protection against oxidative stress and prevents cell and tissue injury [17, 21, 22], and deletion of the Nrf2 gene results in a lupus-like autoimmune nephritis in mice [22, 23]. Disruption of the Nrf2 gene has been shown to result in increased production of IL-1β, TNF-α, and IL-6 in mice [24–26]. Recently, we showed that expression of Nrf2 and its downstream molecules, heme oxygenase-1 and glutathione peroxidase, is reduced in mouse models of diabetic nephropathy, IgA nephropathy, and ASLN, and that these changes can be prevented by treatment with Chinese herbal medicine-derived pure components with Nrf2-mediated anti-oxidant activity [14, 27, 28]. In addition, reactive oxygen species (ROS) can cause NLRP3 inflammasome activation [14, 29], and both oxidative stress and inflammation have been shown to be involved in the development of LN [18, 30] and its progression and deterioration [17, 27].

Citral (3,7-dimethyl-2,6-octadienal), a major active compound found in *Litsea cubeba*, a traditional Chinese herbal medicine, can inhibit activation of macrophages and NF-κB and the production of pro-inflammatory cytokines, including IL-1β [31] and also shows potent antioxidant activity [32, 33]. In a recent study, we demonstrated that the renoprotective effect of Citral results from activation of the Nrf2 antioxidant pathway and inhibition of NF-κB activation in the early stage of adriamycin-induced focal segmental glomerulosclerosis in mice [34] (patented by Taiwan, I463979 and USA, 8,993,637). However, whether Citral has an effect on the accelerated and progressive stage of immune complex-mediated glomerular disorders, such as ASLN, remains to be determined. In this study, we tested the hypothesis that Citral can attenuate an ASLN model in mice and examined the modes of action of its renoprotective effect. We clearly show that by inhibiting the activation signal of NLRP3 inflammasome and increasing Nrf2 anti-oxidative activity, Citral effectively prevented the development of ASLN.

## Methods

### Preparation of Citral (3, 7-dimethyl-2-7-octadienal)

Citral was isolated from fruits of *L. cubeba,* a traditional Chinese herbal medicine as described previously (purity ≥96 %) [34].

### Mouse ASLN model and experimental protocol

A mouse ASLN model was used in which female NZB/Wf1 mice (8 weeks old) were injected intraperitoneally with either saline or 0.8 μg/g body weight of lipopolysaccharide (LPS) (Sigma-Aldrich, St. Louis, MO, USA) in saline twice weekly for 5 weeks as described previously [27]. Starting 2 days before LPS/saline injection, Citral (200 mg/kg of body weight) or vehicle (corn oil) was administered daily by gavage throughout the study. The mice were sacrificed at the end of week 5 after the start of disease induction. All animal experiments were performed with the approval of the Institutional Animal Care and Use Committee of The National Defense Medical Center, Taiwan, and were conducted in accordance with national guidelines.

### Clinical and pathological evaluation

Urine samples were collected in metabolic cages for 6 h at the end of each week, and urinary albumin and creatinine (Cr) were measured as described previously [35]. Serum samples were collected at the end of each week to measure levels of blood urea nitrogen (BUN) and Cr as described previously [35]. At the end of the study, renal pathology and scoring of glomerular proliferation, fibrinoid necrosis, neutrophil infiltration, crescent formation, and peri-glomerular inflammation was performed on 50 randomly sampled glomeruli [9], and a glomerulonephritis activity score (range 0–24) was calculated as described previously [36].

### Immunofluorescence, immunohistochemistry, and terminal deoxynucleotidyl transferase dUTP nick end labeling (TUNEL)

Frozen sections of renal tissues were stained with fluorescein isothiocyanate (FITC)-conjugated antibodies against IgG or C3 (Cappel Lab. Inc., Cochranville, PA, USA) as described previously [37] and semiquantitative analysis of the total immunofluorescence intensity performed as described previously [38]. Formalin-fixed and paraffin-embedded renal sections were incubated with antibodies against CD3 (pan-T cell), or F4/80 (monocytes/macrophages) (both from Serotec, Kidlington, UK), followed by biotinylated second antibodies, and avidin-biotin-peroxidase complex (both from Dako Denmark A/S, Glostrup, Denmark) as described previously [28].

The TUNEL assay was used to detect apoptosis in renal sections using an ApopTag Plus Peroxidase in Situ Apoptosis Detection kit (Chemicon International, Inc., Billerica, MA, USA) according to the manufacturer’s instructions. Numbers of CD3-, F4/80-, or TUNEL-positive cells were determined using PAX-it software as described previously [28].

### Serum levels of autoantibody

Serum anti-dsDNA antibodies were measured using an anti-dsDNA ELISA kit (Alpha Diagnostic, TX, USA) according to the manufacturer’s instructions. The absorbance at 450 nm was measured using an ELISA plate reader (Bio-Tek, Winooski, VT, USA).

### Flow cytometry

Isolated splenocytes were double-stained with FITC-conjugated antibodies against mouse CD3 (pan-T cells), CD4, CD8 (T cell subsets), or CD19 (B cell marker) and phycoerythrin (PE)-conjugated anti-mouse CD69 antibodies (H1.2 F3; marker of activated T and B cells) (BD Biosciences, San Diego, CA, USA) and analyzed on a FACSCalibur (BD Biosciences) as described previously [37].

For intracellular staining of IFN-γ or IL-4, splenocytes were cultured for 5 h in 24-well microtiter plates in the presence or absence of phorbol myristate acetate, ionomycin, and monensin (all from Sigma-Aldrich). They were then stained for 30 minutes on ice with FITC-conjugated anti-mouse CD3 antibodies (BD Biosciences), fixed in 1 % paraformaldehyde (Sigma-Aldrich), and re-suspended in permeabilization buffer, as described previously [37], then intracellular cytokines was stained for 30 minutes on ice with PE-conjugated antibodies against IFN-γ or IL-4 (BD Biosciences), followed by flow cytometric analysis using a FACSCalibur (BD Biosciences).

### Renal levels of ROS

Renal ROS levels were estimated using a chemoluminescence assay for superoxide anion, the results being presented as reactive luminescence units (RLU) per 15 minutes per milligram dry weight (i.e., RLU/15 min/mg dry weight) as described previously [9].

### Renal activities of NF-κB and cytoplasmic caspase-1 activity and serum levels of IL-1β and prostaglandin E2 (PGE2)

Renal cytoplasmic and nuclear proteins were extracted using a nuclear extract kit (Active Motif, Carlsbad, CA, USA) according to the manufacturer’s instructions. Nuclear NF-κB p65 activation was quantified using an ELISA-based TransAM NF-κB kit (Active Motif) according to the manufacturer’s protocol. Caspase-1 activity in the cytoplasmic fraction was measured using caspase-1 activity kits (R&D Systems, Minneapolis, MN, USA) according to the manufacturer’s instructions, and is presented as a value relative to the protein concentration. Serum levels of IL-1β or PGE2 were measured using commercial ELISA kits (both from R&D Systems), according to the manufacturer’s instructions.

### Western blots to estimate levels of nuclear Nrf2 and cytosolic NAD(P)H oxidase subunit p47^phox^, cyclooxygenase-2 (COX-2), NLRP3, and IL-1β in the kidney

Cytoplasmic and nuclear proteins extracted from renal tissues as described above were used for Western blot analysis using antibodies against Nrf2, p47^phox^, COX-2, or IL-1β (all from Santa Cruz Biotechnology, Santa Cruz, CA, USA) or NLRP3 (Enzo Life Sciences Inc, Farmingdale, NY, USA). Antibodies against Lamin A (nuclear proteins) or β-actin (cytosolic proteins), both from Santa Cruz, were used as internal controls.

### In vitro *studies*

#### Effect of Citral on LPS/ATP-induced IL-1β secretion by primary peritoneal macrophages

Peritoneal macrophages, elicited by an intraperitoneal (IP) injection in 8-week-old female NZB/Wf1 mice with 4 % sterile thioglycollate medium as described previously [14], were harvested and grown in RPMI 1640 medium supplemented with 2 mM l-glutamine, 100 U/ml of penicillin, 100 μg/ml of streptomycin, 2.5 μg/ml of amphotericin B, and 10 % heat-inactivated fetal calf serum (all from Gibco, Grand Island, NY, USA) at 37 °C in a 5 % CO_2_ incubator. The cells (2.5 × 10^6^ in 500 μl of medium) were incubated for 30 minutes with Citral or saline, then LPS (Sigma-Aldrich; final concentration 1 μg/ml) was added for 5.5 h. The cells were then incubated for 30 minutes with addition of ATP (Sigma-Aldrich; 5 mM) for 30 minutes, then IL-1β levels in the culture medium were measured using an ELISA kit (R&D Systems), according to the manufacturer’s instructions.

#### Effect of Citral on NLRP3 inflammasome activation in a macrophage cell line

The murine macrophage cell line J774A.1 was purchased from the American Type Culture Collection (Rockville, MD, USA). The cells (2 × 10^6^ in 2 ml of medium) were (1) incubated for 30 minutes with or without Citral, then LPS (final concentration 1 μg/ml) or saline was added for 5.5 h, and incubated for 30 minutes with 5 mM ATP or saline, or (2) incubated for 5.5 h with LPS (1 μg/ml) or saline and the cells were washed with saline, then Citral or saline was added for 30 minutes, followed by addition of 5 mM ATP or saline for 30 minutes. In both assays, IL-1β levels in the culture medium were then measured using an ELISA kit (R&D Systems), while levels of NLRP3, activated caspase-1 (p10), or pro-caspase-1 (p45) in the cells were measured by western blotting, as described previously [28].

### Data analysis

For animal experiments, the results are presented as the mean ± standard error of the mean (SEM) and comparisons between two groups were performed using Student’s *t* test except in the case of differences in urinary albumin/Cr ratios, which were examined using one-way analysis of variance (ANOVA). For experiments using cultured macrophages, all values are given as the mean ± SD, and data analysis was performed using one-way ANOVA with subsequent Scheffé test. A *p* value <0.05 was considered statistically significant.

## Results

### Citral improves albuminuria and renal function

**Fig. 1:**
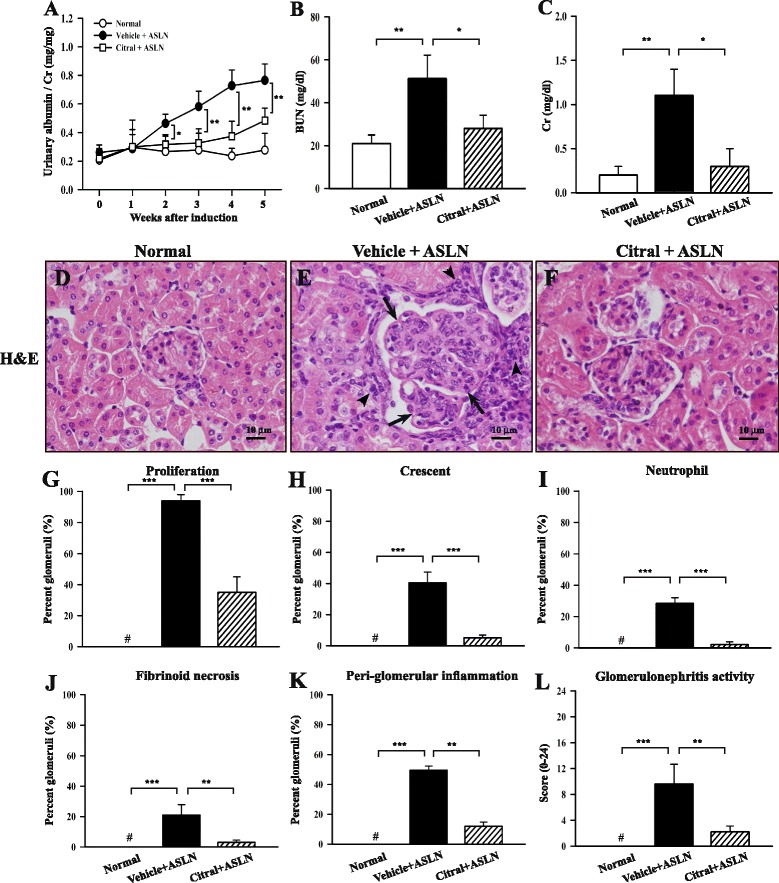
Effects of Citral on clinical, pathological features and renal lesions in mouse model. **a** Time-course of changes in the urine albumin/creatinine ratio. **b** Serum blood urea nitrogen (BUN) levels; **c** serum creatinine (*Cr*) levels. **d**-**f** Kidney histopathological evaluation by H&E staining; **g**-**k** percentage of glomeruli affected by the indicated parameter. **l** Scoring of glomerulonephritis activity. Original magnification × 400. *Bars* show mean ± standard error of the mean for 7 mice per group; *p <0.05, **p <0.01, ***p <0.005*,* ^#^not detectable. *ALSN* accelerated and severe lupus nephritis

A progressive type of mouse ASLN model was used in which female NZB/Wf1 mice (8 weeks old) were injected IP with LPS twice weekly for 5 weeks, and starting 2 days before LPS injection were given Citral or vehicle daily by gavage until the end of the study. As shown in Fig. 1a, vehicle + ASLN mice developed albuminuria which began at the end of week 2 and increased up to the end of the study. However, in Citral-treated ASLN (Citral + ASLN) mice, this effect was markedly inhibited. Similarly, improved renal function was seen at the end of week 5 in Citral + ASLN mice compared to vehicle + ASLN mice as demonstrated by serum levels of BUN (Fig. 1b) and Cr (Fig. 1c) (both *p* <0.05).

### Citral prevents severe renal injury and moderates systemic T cell activation

**Fig. 2:**
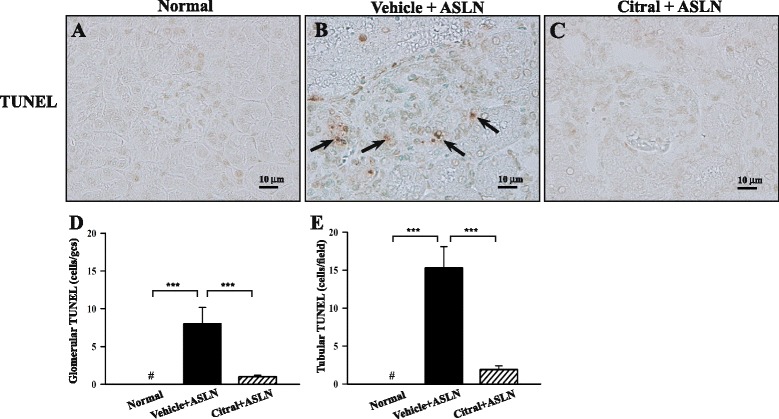
Effects of Citral on renal apoptosis in a mouse model. **a**-**c** Apoptosis in the kidney detected by terminal deoxynucleotidyl transferase-mediated dUTP nick-end labeling (TUNEL). **d**-**e** Scoring of apoptotic cells in the glomerulus as cell number/glomerular cut section (*gcs*) (**d**) or in the tubules as cell number per field (**e**). Original magnification × 400. *Bars* show mean ± standard error of the mean for 7 mice per group; ***p <0.005*,* ^#^not detectable. *ALSN* accelerated and severe lupus nephritis

Light microscopy showed that compared to normal control mice (Fig. 1d), vehicle + ASLN mice had severe renal pathological lesions, including intrinsic cell proliferation (Fig. 1e, g), cellular crescents (Fig. 1e, h), neutrophil influx (Fig. 1e, i), fibrinoid necrosis (Fig. 1e, j) in the glomerulus, and peri-glomerular infiltration of mononuclear leukocytes (Fig. 1e, k). Again, the severity of these renal lesions was markedly reduced in Citral + ASLN mice (all *p* <0.01) (Fig. 1f,g-k), although they still had very mild renal pathological changes. We also calculated a histopathology score, the glomerulonephritis activity score [36], developed to determine glomerulonephritis activity for the assessment of LN in humans, and found that this was markedly increased in vehicle + ASLN mice compared to normal controls and significantly lower in Citral + ASLN mice (Fig. 1l). In addition, as shown in Fig. 2, apoptosis was frequently seen in the kidneys of vehicle + ASLN mice and was significantly decreased in the glomeruli and renal tubules of Citral + ASLN mice, in which there were only a few apoptotic figures.

Immunofluorescence studies demonstrated marked deposits of IgG and C3 in the glomeruli of vehicle + ASLN mice compared to normal control mice (total intensity score 204 ± 18 vs. 31 ± 8, *p* <0.005), which were still present in the glomeruli of the Citral + ASLN mice (total intensity score 211 ± 23). Although significantly increased serum levels of anti-dsDNA antibody were seen in vehicle + ASLN (1.6 ± 0.2 optical density (OD) vs. 0.6 ± 0.1 OD, *p* <0.01) and Citral + ASLN (1.4 ± 0.3 OD vs. 0.6 ± 0.1 OD, *p* <0.01) mice compared to normal control mice, there was no significant difference in serum levels of the autoantibody between vehicle + ASLN and Citral + ASLN mice. The results obtained from both experiments suggest that Citral did not have inhibitory effects on humoral immune response in the mice at the dose used throughout the study.

**Fig. 3:**
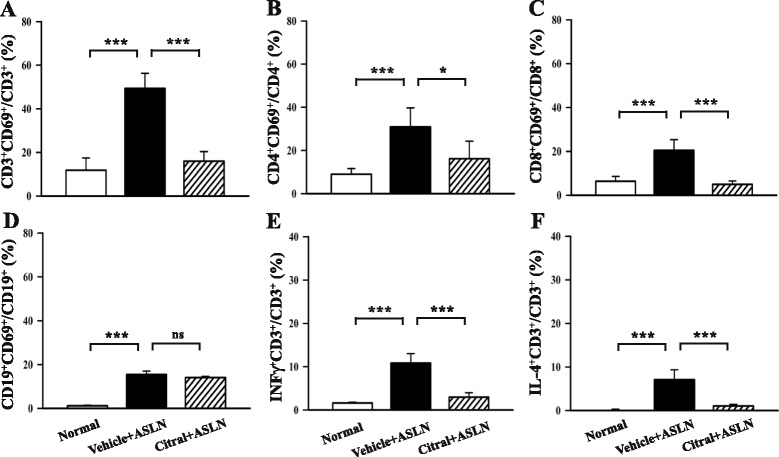
Effects of Citral on systemic T cell and B cell activation and intracellular staining of T lymphocytes from the spleen for IFN-γ or IL-4 in a mouse model. **a**-**d** Percentage of systemic CD3^+^ T cells (**a**), CD4^+^ T cells (**b**), CD8^+^ T cells (**c**), or CD19^+^ B cells (**d**) expressing the activation marker CD69. **e** and **f** Percentage of spleen CD3^+^ T cells expressing IFN-γ^+^ (**e**) or IL-4^+^ (**f**). *Bars* show mean ± standard error of the mean for 7 mice per group; *p <0.05, ***p <0.005, *ns* not significant. *ALSN* accelerated and severe lupus nephritis

Flow cytometric analysis of spleen cells showed that activation of CD3^+^ T cells (Fig. 3a), CD4^+^ T cells (Fig. 3b), and CD8^+^ T cells (Fig. 3c), as shown by the percentage of such cells expressing the activation marker CD69, was significantly increased in vehicle + ASLN mice compared to normal controls, and this effect was again markedly inhibited in Citral + ASLN mice (Fig. 3a-c). In contrast, although activation (CD69 positivity) of B cells (CD19^+^ cells) was significantly increased in vehicle + ASLN mice compared to normal controls, there was no significant difference between the vehicle + ASLN and Citral + ASLN mice (Fig. 3d). The percentage of total T cells (CD3^+^ cells) expressing IFN-γ (Fig. 3e) or IL-4 (Fig. 3f) was greatly increased in vehicle + ASLN mice compared to normal controls, and this effect was markedly inhibited in Citral + ASLN mice.

### Citral inhibits NLRP3 inflammasome activation

As activation of the NLRP3 inflammasome in macrophages has been shown to be involved in the progression of spontaneous SLE in NZB/Wf1 mice [9], we performed several experiments to examine the mechanism of action of Citral in ASLN mice and in vitro cultures.

#### Animal model

**Fig. 4:**
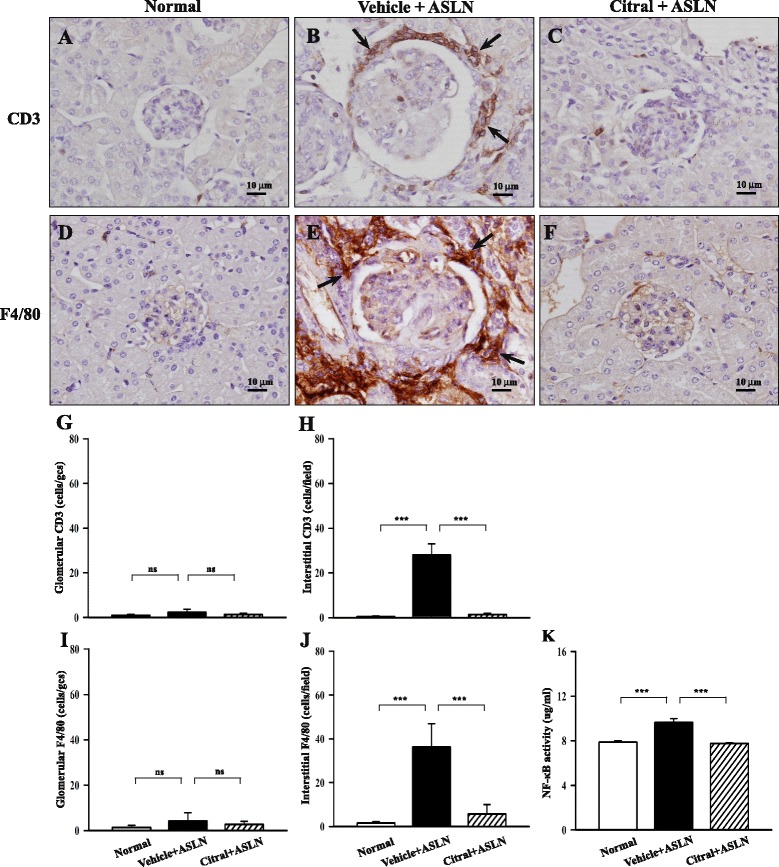
Effects of Citral on renal infiltration by monocytes/macrophages and T cells and NF-κB activation in the kidney of a mouse model. Detection of CD3^+^ T cells (**a**-**c**) and F4/80^+^ monocytes/macrophages (**d**-**f**) by immunohistochemistry. Original magnification × 400. *Arrows* indicate positively stained cells. Scoring of CD3^+^ cells (**g** and **h**) or F4/80^+^ cells (**i** and **j**) in the glomerulus (**g** and **i**) or interstitium (**h** and **j**). **k** Nuclear NF-κBp65 activity. *Bars* show mean ± standard error of the mean for 7 mice per group; ***p <0.005, *ns* not significant. *ALSN* accelerated and severe lupus nephritis

Significant renal infiltration of T cells (CD3^+^) was seen at the end of week 5 in vehicle + ASLN mice compared to normal control mice and this effect was markedly inhibited in Citral + ASLN mice (Fig. 4a-c, g, h). In addition, in vehicle + ASLN mice there was significant infiltration of macrophages (F4/80^+^) into the kidney and this effect was also significantly decreased in Citral + ASLN mice (Fig. 4d-f, i, j). Renal nuclear NF-κB p65 activity was also significantly increased in vehicle + ASLN mice compared to normal controls and was also significantly inhibited in Citral + ASLN mice (Fig. 4k).

**Fig. 5:**
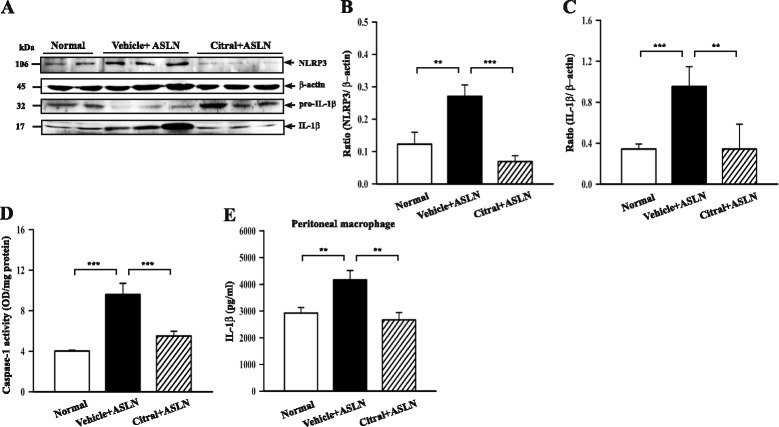
Effect of Citral on NLRP3 inflammasome activation in the kidney of the animal model, and on in vitro lipopolysaccharide (LPS) + ATP-induced IL-1β secretion by naïve peritoneal macrophages. **a** Representative western blots for cytosolic levels of NLRP3 and IL-1β in the kidney, with β-actin as the loading control. **b** and **c** Semiquantitative analysis of the NLRP3/β-actin ratio and IL-1β/β-actin ratio. **d** Cytosolic caspase-1 activity measured by ELISA and expressed relative to the protein content. **e** Effect of Citral pretreatment of peritoneal macrophages from naïve mice on in vitro LPS + ATP-induced IL-1β secretion. Peritoneal macrophages were incubated with Citral or saline for 30 minutes, then LPS was added for 5.5 h, when the cells were incubated for 30 minutes with ATP. The controls were incubated with saline at each stage. *Bars* show mean ± standard error of the mean for 7 mice per group; **p <0.01, ***p <0.005. *ALSN* accelerated and severe lupus nephritis

Western blotting showed increased NLRP3 expression in the kidneys of vehicle + ASLN mice compared to normal control mice (*p* <0.01) and this effect was prevented by Citral treatment (*p* <0.005) (Fig. 5a, b). Vehicle + ASLN mice also expressed significantly higher renal IL-1β levels (Fig. 5a, c) and renal caspase-1 activity than normal control mice (Fig. 5d) and Citral administration resulted in a significant reduction in both (both *p* <0.01). In addition, significantly increased serum levels of IL-1β were observed in vehicle + ASLN mice, compared to normal control mice (86.2 ± 15.0 pg/ml vs. 46.3 ± 8.5 pg/ml; *p* <0.05), and Citral administration resulted in decreased serum levels of IL-1β, although there was no statistical significance (86.2 ± 15.0 pg/ml vs. 61.8 ± 9.2 pg/ml; *p* = 0.06).

#### In vitro studies of the effect of Citral on LPS + ATP-induced IL-1β secretion by naïve peritoneal macrophage cultures or the mouse macrophage cell line

Full activation of the NLRP3 inflammasome requires both a priming signal (e.g., LPS) and an activation signal (e.g., ATP) [14, 39]. In the first study, peritoneal macrophages from untreated 8-week-old female NZB/Wf1 mice were incubated in vitro for 30 minutes with Citral or saline, followed by addition of LPS for 5.5 h, then were incubated with ATP for 30 minutes, when IL-1β levels in the culture medium were measured by ELISA; controls were cells incubated at each stage with saline. The results showed that Citral completely blocked LPS + ATP-induced IL-1β production (Fig. 5e).

**Fig. 6:**
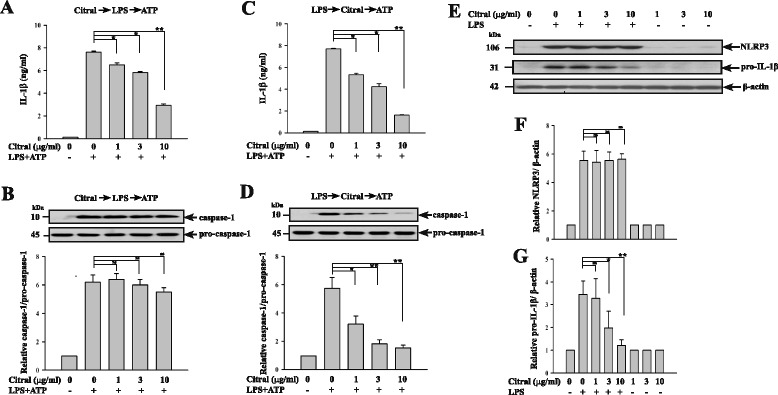
In vitro effect of Citral on NLRP3 inflammasome activation in J774A.1 macrophages. **a** IL-1β secretion by lipolysachharide (*LPS*) + ATP-activated macrophages. **b** Western blots showing caspase-1 activation in LPS + ATP-activated macrophages. **c** ATP-induced IL-1β secretion by LPS-primed macrophages. **d** ATP-induced caspase-1 activation in LPS-primed macrophages. **e** Representative western blots showing the effect of treatment with different concentrations of Citral for 30 minutes before addition of LPS for 6 h on NLRP3 and pro-IL-1β levels in J774A.1 macrophages. **f** and **g** Semiquantitative analysis of the NLRP3/β-actin ratio (**f**) and pro-IL-1β/β-actin ratio (**g**). The data are expressed as the mean ± SD for three separate experiments; the histogram shows the quantification expressed as the mean *±* SD*;* *p <0.05, **p <0.01*, ns* not significant

In the second study, we examined the effect of Citral on each step of LPS/ATP-induced NLRP3 inflammasome activation in the mouse macrophage cell line J774A.1. In the first experiment, control J774A.1 macrophages were left untreated, while others were incubated with or without different concentrations of Citral for 30 minutes before addition of LPS for 5.5 h, and incubation with ATP for 30 minutes, then IL-1β secretion was measured by ELISA and caspase-1 generation by western blotting. The results showed that Citral significantly inhibited IL-1β secretion in a dose-dependent manner (Fig. 6a), but did not affect the generation of active caspase-1 (Fig. 6b). In the second experiment, we incubated the cells with LPS for 5.5 h, washed with saline, before addition of vehicle or different concentrations of Citral for 30 minutes, followed by ATP stimulation for 30 minutes, and found that Citral inhibited both IL-1β secretion (Fig. 6c) and caspase-1 activation (Fig. 6d) in a dose-dependent manner. These results demonstrate that Citral inhibits the production and secretion of IL-1β through activation of the NLRP3 inflammasome. We also tested its ability to inhibit expression of both NLRP3 and pro-IL-1β (IL-1β precursor) proteins in LPS-activated J774A.1 cells by incubating the cells with different concentrations of Citral for 30 minutes before addition of LPS for another 6 h, and found that Citral had no effect on LPS-induced NLRP3 expression (Fig. 6e, f), but inhibited LPS-induced pro-IL-1β expression in a dose-dependent manner (Fig. 6e, g).

### Citral inhibits the increase in ROS, NAD(P)H oxidase subunit p47^phox^, COX-2, or PGE2 levels, and it enhances nuclear Nrf2 levels

**Fig. 7:**
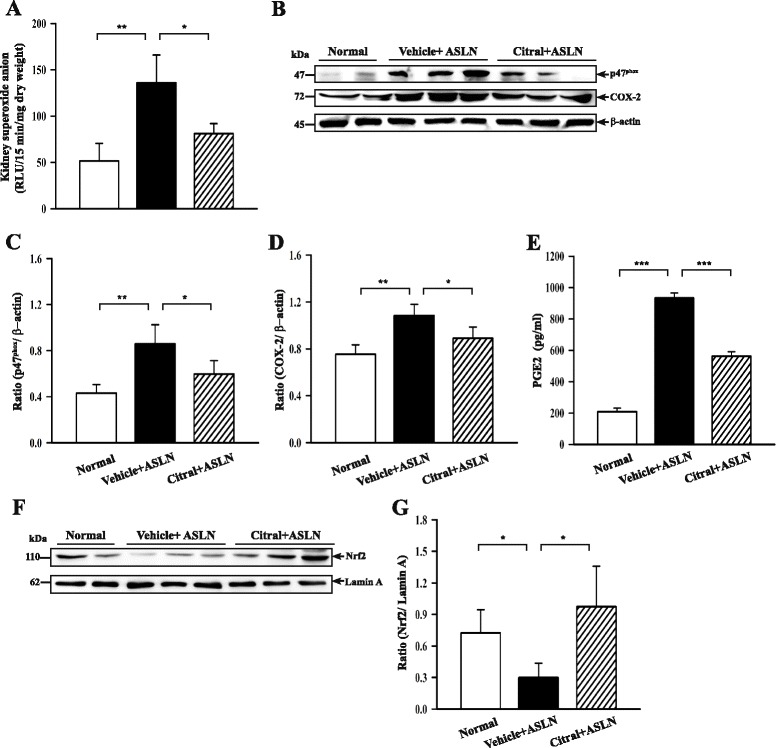
Effect of Citral on renal levels of superoxide anion, cytoplasmic NAD(P)H oxidase subunit p47^phox^ and cyclooxygenase-2 (*COX-2*), nuclear nuclear factor E2-related factor 2 (*Nrf2*), and serum prostaglandin E2 (*PGE2*) in mouse model. **a** Reactive oxygen species levels in renal tissues expressed as reactive luminescence units (RLU)/15 min/mg dry weight. **b** Representative western blots for cytosolic levels of p47^phox^ and COX-2, with β-actin as the loading control. **c** and **d** Semiquantitative analysis of the p47^phox^/β-actin ratio (**c**) and the COX-2/β-actin ratio (**d**). **e** Serum PGE2 levels measured by ELISA. **f** Representative western blots for nuclear levels of Nrf2. with Lamin A as the loading control. **g** Semiquantitative analysis of the Nrf2/Lamin A ratio. *Bars* show mean ± standard error of the mean for 7 mice per group; *p <0.05, **p <0.01, and ***p <0.005. *ASLN* accelerated and severe lupus nephritis

Citral has been shown to be an antioxidant in inflammatory diseases [14, 40] and inhibits LPS-induced ROS production in macrophages [34, 40]. As shown in Fig. 7a, renal levels of superoxide anion were significantly increased in vehicle + ASLN mice compared to normal controls (*p* <0.01) and this effect was significantly inhibited in Citral + ASLN mice (*p* <0.05). Renal cytosolic p47^phox^ levels in vehicle + ASLN mice were also greatly increased compared to normal control mice (*p* <0.01), and this effect was significantly decreased by Citral treatment (*p* <0.05) to levels similar to those in normal control mice (Fig. 7b, c). Next, we measured renal cytosolic COX-2 levels and serum levels of PGE2, a downstream molecule of COX-2, and found that renal COX-2 levels (Fig. 7b, d) and serum PGE2 levels (Fig. 7e) were significantly increased in vehicle + ASLN mice compared to normal control mice (both *p* <0.01), and both effects were significantly inhibited in Citral + ASLN mice (both *p* <0.05). We then evaluated nuclear levels of Nrf2 in renal tissues, as this transcription factor triggers an antioxidant pathway in renal inflammation and fibrosis [27, 28]. As shown in Fig. 7f and g, vehicle + ASLN mice had significantly lower nuclear levels of Nrf2 than normal control mice (*p* <0.05) and this effect was significantly decreased in Citral + ASLN mice (*p* <0.05).

## Discussion

In the present study, as persistent inflammatory episodes and oxidative stress are implicated in both the development and progression/deterioration of LN [20, 27], we examined whether Citral could alleviate the clinical manifestations and pathological changes in an ASLN model in NZB/Wf1 mice. Our results showed that Citral inhibited the development of ASLN, as demonstrated by reduced albuminuria, improved renal function, and improved renal histopathology in intrinsic cell proliferation, cellular crescents, neutrophil influx, fibrinoid necrosis in the glomerulus, and peri-glomerular infiltration of mononuclear leukocytes, and the glomerulonephritis activity score. Mechanistic studies showed that inhibition of the activation signal of NLRP3 inflammasome and enhanced activation of Nrf2 antioxidant signaling were involved in the major mechanisms of action for the renoprotective effects of Citral.

Bachiega et al*.* (2011) [31] demonstrated that Citral reduces LPS-induced IL-1β secretion in macrophages. We further examined the inhibitory effect of Citral on NLRP3 inflammasome activation was verified by naïve peritoneal macrophages from untreated mice incubated with Citral or saline, then tested for LPS/ATP-induced IL-1β secretion (Fig. 5e). Citral significantly reduced both IL-1β secretion (Fig. 6c) and caspase-1 activation (Fig. 6d) when added to cultures of J774A.1 macrophages after LPS priming, but before ATP stimulation, suggesting that it reduces IL-1β secretion by inhibiting ATP-mediated caspase-1 activation. In addition, addition of Citral before LPS priming also caused reduced IL-1β secretion (Fig. 6a), but did not reduce caspase-1 activation (Fig. 6b). Although Citral did not affect NLRP3 expression in LPS-activated macrophages (Fig. 6e, f), it reduced LPS-induced pro-IL-1β expression (Fig. 6e, g). We therefore infer that the Citral-mediated decrease in IL-1β secretion in LPS-primed macrophages involves downregulation of pro-IL-1β production. Collectively, our in vitro data indicate that Citral inhibits IL-1β secretion through: (1) reducing IL-1β precursor (pro-IL-1β) protein expression (Fig. 6e) and (2) inhibiting ATP-induced caspase-1 activity (Fig. 6d). These results indicate that Citral inhibits both LPS-mediated pro-IL-1β expression and ATP-mediated activation signal of NLRP3 inflammasome.

Interestingly, the fact that Citral administration *in vivo* resulted in reduced renal NLRP3 expression in Citral-treated mice (Citral + ASLN mice) suggests that other molecular pathways may be involved in the mechanism of action of Citral in this mouse model of ASLN. In this regard, for instance, binding of IL-1β to IL-1 receptors was found to promote production of COX-2 [41]. In our recent report, COX-2 promotes NLPR3 activation and IL-1β protein secretion [39]. In the present study, we demonstrated that reduced renal COX-2 levels were seen in Citral + ASLN mice compared to vehicle + ASLN mice (Fig. 7b, d). The data suggest the COX-2-mediated activation of NLRP3 inflammasome may be one of attributed pathways responsible for the renoprotective effects of Citral. It should be noted, however, that to determine whether the NLRP3 inflammasome-associated molecules and related signaling pathways are crucial in the mechanisms of Citral for its renoprotective effects, the ASLN model should be used in mice deficient in each of the several molecules or with neutralizing antibodies, and all these tests deserve further investigation. Another issue is whether the effect of Citral is specific for the NLRP3-inflammasome-mediated IL-1β production. Other inflammasomes, such as AIM2 and NLRC4 inflammasomes, deserve further evaluation of the renoprotective effects of the compound.

Finally, Citral prevented kidney inflammation and resultant fibrosis n this severe LN model by inhibiting the systemic immune response. Abnormal systemic function of T and B cells in innate and adaptive immune responses is involved in the canonical pathophysiological pathways underlying the development and progression of LN [4, 6]. In the present study, although we failed to show any inhibitory effects of Citral on B cell activation or on immune deposits in the kidneys, Citral administration markedly inhibited CD4^+^ T cell activation (Fig. 3b) and the production of IFN-γ by these cells (Fig. 3e). Importantly, T helper (Th)1 cells can promote the activation of macrophages by producing IFN-γ, and macrophages are involved in NLRP3 inflammasome activation, which may provide IL-1β signaling to activate T cells [42, 43]. The beneficial effect of Citral administration in this severe LN model may involve an effect on Th cell responses. This potential mode of action of Citral in its renoprotective effects is worth further investigation.

Blockade of ROS production in studies involving the use of chemical scavengers of ROS, pharmacological inhibitors of NAD(P)H oxidase, or siRNA-mediated knockdown of p22^phox^ NAD(P)H oxidase has been shown to inhibit NLRP3 inflammasome activation in response to a wide range of stimuli [14, 44]. Our previous study showed that inflammasome activation is mediated by ROS by using ROS inhibitors [45] Taken together, we infer that reduced ROS generation by Citral may explain the resultant decreased pro-IL-1β protein levels by the compound. Furthermore, we showed that increased ROS production was seen in the kidney in ASLN mice and that Citral activated Nrf2 in ASLN mice, supporting the ideas that oxidative stress contributes to the pathogenesis of ASLN and that enhanced activation of the Nrf2 antioxidant pathway prevents the renal damage associated with severe LN. NLRP3 expression is tightly controlled by the activity of multiple signaling receptors, and activation of NF-κB results in activation of the NLRP3 inflammasome and resultant IL-1β production [46]. In the present study, we showed that increased production of ROS (Fig. 7a) and increased NF-κB activation (Fig. 4k) were seen in the kidney of vehicle + ASLN mice and these effects were significantly inhibited by Citral. This finding is in agreement with our previous report that Citral administration can markedly inhibit NF-κB p65 activation in the kidney cortex in a renal inflammation and fibrosis model in mice [34]. Furthermore, renal NLRP3 levels in vehicle + ASLN mice were increased and renal posttranslational processing of caspase-1 and IL-1β promoted, all suggestive of NLRP3 inflammasome activation, and these effects were inhibited in Citral + ASLN mice (Fig. 5). However, a decrease of NLRP3 levels and IL-1β and increases in Nrf2 levels in kidneys after Citral treatment compared to vehicle treatment (Figs. 5a and 7g), which may result from the reduced recruitment of macrophages into the kidney after treatment, but this may be part of the mechanisms involved in these proteins in the protective effects of Citral. A selective Nrf2 agonist, bardoxolone, has been found to enhance the magnitude of proteinuria in a rat model of diabetic nephropathy [47], but was found to be beneficial in the prevention and therapy of tissue injury in an animal model of cancer [48, 49]. Further studies are needed to resolve these conflicting results and determine the mechanistic pathways involved.

## Conclusion

Citral alleviates the mouse ASLN model by inhibition of the activation signal of NLRP3 inflammasome and enhanced activation of Nrf2 antioxidant signaling.

## Abbreviations

ANOVA: analysis of variance
ASLN: accelerated and severe lupus nephritis
BUN: blood urea nitrogen
COX-2: cyclooxygenase-2
Cr: creatinine
ELISA: enzyme-linked immunosorbent assay
FITC: fluorescein isothiocyanate
IFN: interferon
IL: interleukin
IP: intraperitoneal
LN: lupus nephritis
LPS: lipopolysaccharide
NF-κB: nuclear factor kappa-light-chain-enhancer of activated B cells
NLRP3: NACHT, LRR and PYD domains-containing protein 3
Nrf2: nuclear factor E2-related factor 2
OD: optical density
PE: phycoerythrin
PGE2: prostaglandin E2
RLU: reactive luminescence units
ROS: reactive oxygen species
SEM: standard error of the mean
SLE: systemic lupus erythematosus
TUNEL: terminal deoxynucleotidyl transferase dUTP nick end labeling
